# A comparative structural bioinformatics analysis of inherited mutations in β-D-Mannosidase across multiple species reveals a genotype-phenotype correlation 

**DOI:** 10.1186/1471-2164-12-S3-S22

**Published:** 2011-11-30

**Authors:** Thi Huynh, Javed Mohammed Khan, Shoba Ranganathan

**Affiliations:** 1 Department of Chemistry and Biomolecular Sciences and ARC center of excellence in Bioinformatics, Macquarie University, NSW 2109, Australia; 2 Department of Biochemistry, Yong Loo Lin School of Medicine, National University of Singapore, 8 Medical Drive, Singapore 117597

## Abstract

**Background:**

Lysosomal β-D-mannosidase is a glycosyl hydrolase that breaks down the glycosidic bonds at the non-reducing end of N-linked glycoproteins. Hence, it is a crucial enzyme in polysaccharide degradation pathway. Mutations in the *MANBA* gene that codes for lysosomal β-mannosidase, result in improper coding and malfunctioning of protein, leading to β-mannosidosis. Studying the location of mutations on the enzyme structure is a rational approach in order to understand the functional consequences of these mutations. Accordingly, the pathology and clinical manifestations of the disease could be correlated to the genotypic modifications.

**Results:**

The wild-type and inherited mutations of β-mannosidase were studied across four different species, human, cow, goat and mouse employing a previously demonstrated comprehensive homology modeling and mutational mapping technique, which reveals a correlation between the variation of genotype and the severity of phenotype in β-mannosidosis. X-ray crystallographic structure of β-mannosidase from Bacteroides thetaiotaomicron was used as template for 3D structural modeling of the wild-type enzymes containing all the associated ligands. These wild-type models subsequently served as templates for building mutational structures. Truncations account for approximately 70% of the mutational cases. In general, the proximity of mutations to the active site determines the severity of phenotypic expressions. Mapping mutations to the *MANBA* gene sequence has identified five mutational hot-spots.

**Conclusion:**

Although restrained by a limited dataset, our comprehensive study suggests a genotype-phenotype correlation in β-mannosidosis. A predictive approach for detecting likely β-mannosidosis is also demonstrated where we have extrapolated observed mutations from one species to homologous positions in other organisms based on the proximity of the mutations to the enzyme active site and their co-location from different organisms. Apart from aiding the detection of mutational hotspots in the gene, where novel mutations could be disease-implicated, this approach also provides a way to predict new disease mutations. Higher expression of the exoglycosidase chitobiase is said to play a vital role in determining disease phenotypes in human and mouse. A bigger dataset of inherited mutations as well as a parallel study of β-mannosidase and chitobiase activities in prospective patients would be interesting to better understand the underlying reasons for β-mannosidosis.

## Background

β-D-mannosidase is a lysosomal exoglycosidase that belongs to the glycosyl hydrolase family. By hydrolyzing the single β-linked mannose residue at the non-reducing end of all N-linked glycoproteins, it serves as the last catalyst in the glycanic moieties degradation pathway of glycoconjugates [1]. Genetic information of β-D-mannosidase is stored in a gene called *MANBA*, which is located on different positions in different species [2]. In human, *MANBA* is located on the long arm of chromosome 4 (4q22-q25) [2]. Mutations in *MANBA* result in β-mannosidosis, a lysosomal storage disorder where the activity of β-mannosidase is reduced or completely demolished [3].

β-mannosidosis is a rare autosomal recessive disease that was first diagnosed in Nubia goat [4], and later in human [3,5] and domestic cow [6]. It is characterized by excessive accumulation of (di)-saccharides or (tri)-saccharides in lysosomes [7], and the phenotypic expressions vary across the three species. In ruminants, all the mutations are homozygous and enzyme defects result in severe neonatal conditions, which are usually lethal without intensive care [8]. The affected animals suffer from inability to rise, tension tremors, facial dysmorphism, slight domed calvaria and prognathism, marrow palpebral fissures and carpal contractures [4,6]. In human, β-mannosidosis can either be homozygous or heterozygous, and the clinical conditions are reasonably milder. The ages of onset vary from infantile to 24 years old. Some typical phenotypes include (but are not limited to) hearing loss, mental retardation, angiokeratomas and facial dysmorphism [3,9]. Although only 21 cases from 17 families are reported to date, the number of incidents can be much higher, as a carrier with mild or non-typical symptoms would never be diagnosed. Despite several attempts to study the genotypes and phenotypes of β-mannosidosis, no correlation between them has been established [7,10].

Studying the effects of mutations on the protein structure provides a better understanding of the clinical manifestations of the disease by enhancing the knowledge of the finer aspects of its pathology. Despite the fact that no 3D structure has been solved for mammalian β-mannosidase, such an approach has been proved adequate by Khan and Ranganathan[11] to demonstrate the pathological role of mutations. Therefore, we have employed structural bioinformatics to examine how changes at the genetic level affect the phenotypic expression in β-mannosidosis.

Mutational information was obtained from OMIA (Online Mendelian Inheritance in Animals) [12], OMIM (Online Mendelian Inheritance in Man) [13] and published literature. Among a total of 14 disease-associated mutations reported in human, majority (7) are nonsense mutations [7], with only one case of insertion [14] and two cases of deletions [9,15] being identified to date. The nonsense, insertion and deletion mutations cause truncations at the protein level. Four missense mutations leading to substitutions at the protein level have also been identified in human. In cow and goat, only 1 mutation was identified for each species, both of which were nonsense mutations [8,16]. The nonsense mutation in goat however, is similar to the one reported in human, both of which cause truncation of the protein chain at the same residue (W466). Though springbok was listed in OMIA for β-mannosidosis, it was excluded from this study due to lack of evidence on whether α- or β-mannosidase deficiencies were responsible for the observed phenotypes [17]. On the other hand, wild-type β-mannosidase from mouse was included in this project despite not being listed on OMIA, since a gene-knockout model of mouse was recently used for β-mannosidosis study in human [18].

An X-ray structure of β-mannosidase from *Bacteroides thetaiotaomicron* (Protein Data Bank [19] – PDB ID: 2JE8) [20] was selected as the template for 3D model building, since no mammalian structure of the enzyme is currently available. The structure of β-mannosidase from *Bacteroides thetaiotaomicron* has been solved at a good resolution of 1.70 Å and R-free of 0.188 [20]. Being so distant from mammalian enzymes in the phylogenic tree, bacterial β-mannosidase only shares an average of 35% sequence identity with the mammalian enzymes (obtained by performing a NCBI BLASTp [21] search across the PDB). However, the overall 3D structure of a protein, especially that of enzymes, is known to be highly conserved across species [22] with a requirement of only 25% sequence identity for homology model building [23], thereby suggesting that homology modeling can be utilized as an adequate tool for this study. Moreover, all the active site residues of β-mannosidase are conserved among all the species involved in this study. This suggests functional similarities to the enzymes in the different species studied here.

In this paper, we have extended our previously reported structural bioinformatics analysis method [11] to study β-mannosidosis across four species: cow, goat, human and mouse, in order to understand the effect of mutations on β-mannosidase structure and function. Within the scope of this study, splicing events are eliminated. β-mannosidosis is inherited in a homozygous recessive manner in ruminants. However, it can be the result of a single mutation or multiple mutations on different chromosomes in human. Thus, the peripheral effects are greatly diverged making it extremely important to conduct an in-depth investigation to understand the genotype-phenotype correlation for this disease. This disease can be comparatively well studied as it occurs in different species. This provides us with an evolutionary basis for the conserved regions of the protein sequence, as well as active site conservation, where mutations could result in disastrous consequences. Our comprehensive analysis establishes a genotype-phenotype correlation for β-mannosidosis, contrary to the reports by Riise *et al.*, [7] and Gort *et al.*, [10]. We report five potential mutational hot-spots within the *MANBA* gene sequence where novel mutations could be disease-implicated. Based on the proximity of the mutations within these hot-spots to the protein active site, the severity of the pathological effects or the disease phenotypes: mild, moderate or severe (deduced from known mutations and disease phenotypes), could also be determined. 

## Materials and methods

The principle underlying homology modeling is that protein structure is better preserved during evolution than their sequence [23]. Therefore, homology modeling has been previously applied by our group to derive a strong genotype-phenotype correlation in α-mannosidosis [11]. Motivated by our success with α-mannosidosis and based on the observation that the active sites of β-mannosidase are conserved from *Bacteroides thetaiotaomicron* to mammals, homology modeling has once again been employed as a powerful and reliable method for building 3D structural models of the enzyme for the four species (cow, goat, human and mouse). β-mannosidase from *Bacteroides thetaiotaomicron*[20] has been used as a template for all the mammalian wild-type (WT) models. Unlike α-mannosidase that undergoes cleavage into five distinct chains [11], β-mannosidase is immune to such proteolysis and assembles into a single chain functional protein.

β-mannosidase from *Bacteroides thetaiotaomicron*[20] has two identical chains, chain A and B. Chain B had the most number of residues (841) compared to chain A (837) and the maximum number of associated ligands (nine) [20] amongst the two chains. Therefore, only chain B was used for modeling along with the associated ligands. In order to build high-quality homology models, it is crucial to obtain correct alignments between the amino acid sequence of the template structure and that of the target sequences. Three-dimensional models of the mature wild-type β-mannosidase were constructed for each of the 4 species: cow, goat, human and mouse. These WT models were subsequently utilized as templates to build the respective mutated models. Although the structural model for the mouse WT sequence was built, no mutational models were built for mouse due to the lack of data on mouse mutations causing β-mannosidosis. Therefore, mutational analyses for mouse could not be performed. However, based on the mapping of mutations, mutational co-location from other organisms (cow, goat and human) and their propinquity to the active site of the enzyme, we have predicted mouse mutations that could cause harmful phenotypes. The overall methodology utilized in this investigation is similar to the one previously reported by Khan and Ranganathan [11].

### Data collection

Complete WT lysosomal β-mannosidase protein sequences were obtained from the UniProt database [24], one each for bovine (Accession No: Q29444), goat (Accession No: Q95327), human (Accession No: O00462) and mouse (Accession No: Q8K2I4). The template sequence was retrieved from the X-ray crystal structure of Bacteroides thetaiotaomicron β-mannosidase (PDB ID: 2JE8) [20] using MODELLER [25].

### Preliminary assessment

ClustalX [26] was used with default BLOSUM scoring matrices for the alignment of the full-length WT protein sequences of the four species to the extrapolated sequence of the template PDB structure [20]. Prior to alignment, signal peptides (about 19 N-terminal amino acids in mouse and 17 in the other species as annotated by UniProt) were excised from the WT protein sequences, owing to their absence in the mature template protein structure. In view of a few residues missing from the template PDB structure and in order to conserve chain boundaries along with structurally and functionally important residues, manual edition of gaps in the alignment was carried out with careful scrutiny.

### 3D structural modeling

Previously, we have reported the superiority of MODELLER over other homology modeling softwares given the complexities involved in 3D protein structure modeling, especially the inclusion of ligands [11]. Therefore, MODELLER version 7V7 was employed to construct all the homology models used in this study. The models are built upon optimal satisfaction of dihedral angle restraints and spatial constraints that are derived from the alignment of the target sequence and the template structure [27]. These dihedral angle restraints and spatial constraints along with the CHARMM-22 [28] force field enforce proper stereochemistry. For each of the WT and mutated models, five structural models were generated. After performing stereo-chemical quality assessment and structural refinement, three models with the best objective functions, out of the five, were chosen and one model with the lowest current energy amongst these three was subsequently selected for structural analysis. These high-quality WT structures were subsequently used as templates for constructing all the mutational models. For truncation mutations that are caused as a result of a frame-shift in the MANBA gene sequence, WT mRNA sequences were retrieved from the NCBI Gene [29] database and the nucleotide insertion causing the respective frame-shift was manually carried out. Following this, the mutated mRNA sequences were translated into protein sequences, using the DNA Protein Translate tool [30]. Subsequently, the frame with methionine as the first residue was selected for molecular modeling.

**Figure 1:**
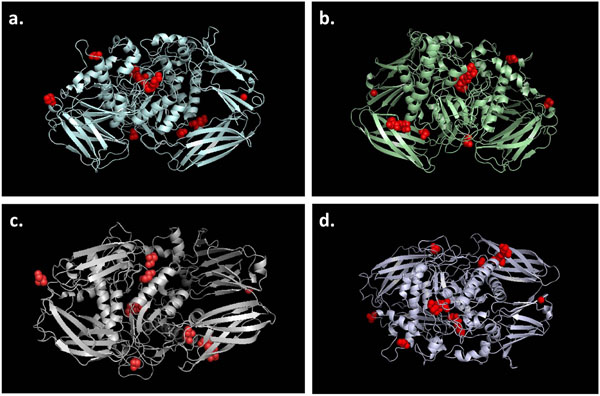
**3D structural models for wild-type (WT) sequences of a. goat, b. bovine, c. human and d. mouse.** The associated ligands are shown as red spheres. All four WT β-mannosidase models are shown in the ribbon representation.

Unlike α-mannosidase where two additional disulfide bridges were built by Khan and Ranganathan [11] for mutational analyses, there is no annotated evidence of disulfide bonds in UniProt for all WT β-mannosidase sequences. Hence, we have not included any disulfide bridges in our models. However, nine ligands existed in the template structure. Thus, to dissect the effect of the observed mutations on the binding of the ligands, we have rebuilt all the ligands in our β-mannosidase models (Figure 1) for the four species by extrapolating their structural coordinates and positional information from the template structure. UniProt also does not provide any information about putative pro-peptide regions for β-mannosidase from all the four species. Thus, we have not incorporated any pro-peptides in our structural models. A number of water molecules were present in the template structure, most of which were surface water molecules of crystallization[20]. However, none of the internal water molecules were within 4Å distance from either the ligands or the active site residues. Moreover, none of them were reported to be functionally critical [20]. Hence, this analysis does not include any water molecules.

Both structural visualization and Root Mean Square Deviation (RMSD) calculation was done using PyMol [31]. The Protein Structure Validation Suite, which incorporates major structural assessment tools such as PROCHECK [32], Verify3D [33] and PDB Validation [34], was used to check the bond-length, bond-angle, residue geometry, stereo-chemical quality and perform volumetric analysis to evaluate the overall quality of each structural model.

## Results and discussion

### High quality 3D models for WT β-mannosidases

The structural models generated by MODELLER undergo multiple stereo-chemical refinements after being selected from a pool of randomized potential starting conformations. The structural quality assessment of the final models was performed using the Protein Structure Validation Suite, as described above. Detailed results of the quality verifications performed on the four WT models are provided in Table 1. RMSD calculation performed by PyMOL returned scores between 0.28 Å and 0.29 Å for all WT models, suggesting excellent quality of the WT structural models. With Z-scores of -1.28 to -1.61 from Verify3D, all four WT models of β-mannosidase were considered to be structurally sound, since an average Z-score of -1.44 is usually obtained for high resolution PDB structures [33].

**Table 1:** Structural quality assessment of wild-type β-mannosidase models

Models	Results of the tests performed						
	
	RMSD (calculated by PyMOL) (Å)	PROCHECK (ramachandran plot statistics)		Verified3D (Z-Score)	Energy (reported by Modeller) (kcal/mol)	PDB validation (deviations from ideal geometry)	
			
		*Residues in most favored regions*	*Residues in allowed regions*			Bond angles (degree)	Bond lengths (Å)
**Cow**	0.292	86.5%	10.8%	-1.61	11577.25	3.3	0.021
**Goat**	0.280	87.8%	8.2%	-1.61	8713.15	3.5	0.020
**Human**	0.295	85.7%	9.9%	-1.44	9281.74	3.2	0.021
**Mouse**	0.295	92.1%	7.1%	-1.28	8503.92	3.3	0.021

PROCHECK predicted that all the WT models had 95.6% - 99.2% of their residues within the conformationally allowed regions, while 85% is the minimum benchmark for high-quality X-ray crystallography structures. Moreover, the PDB Validation software, also used by PDB for 3D structural quality assessment, assigned an average value of 0.02 Å and 3.325° for the bond lengths and bond angles, respectively, within each of the WT models. Hence, all computationally generated models in this investigation are considered to be of good quality and suitable for this structural analysis. High degree of conservation amongst the functionally important domains from the template sequence and the target sequences, together with thorough preliminary assessment have resulted in good quality homology models.

### Structural mapping of mutations

In order to understand the structural location of the mutations and study the impact of mutations on the enzyme’s structure in different species, a mutational map was constructed. Based on multiple sequence alignment and profile alignment of WT human, cow, goat and mouse sequences with that of the template, all the available mutations were mapped to the structural models of β-mannosidase in the context of the active site of the enzyme. Prior to mutational mapping, initial mapping of the secondary structure on the aligned sequences was carried out according to information provided by PDB database on Bacteroides thetaiotaomicron β-mannosidase. (Additional File 1 - Figure S1). Subsequently, positions of the mutations and their relative locations were mapped onto the secondary structures of WT enzymes. 

**Figure 2:**
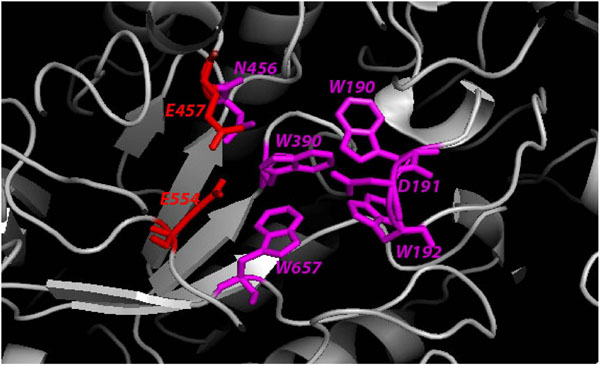
**Human β-mannosidase active site**. The residues that comprise the active site are in stick representation and are labeled. The catalytic nucleophiles (E457 and E554) are coloured red. The binding site residues are in magenta.

Mammalian β-mannosidase is a multi-domain protein with (α/β) TIM barrel being the home of its catalytic nucleophiles, E457 and E554 [20]. All the amino acid residues that take part in the enzymatic activity of β-mannosidase are well conserved from bacteria to human (Additional File 1 - Figure S1). Figure 2 shows the components of β-mannosidase active site in human. The W190, D191 and W192 motif belongs to an extended loop of the first beta-sheet that projects towards the catalytic center. Together with W390 and W657, both of which are also located within loops adjacent to E457, the W190, D191 and W192 residues provide a firm grip to hold substrates into the correct positions for catalysis [20]. All these highly conserved amino acids give the active site of β-mannosidase a deep pocket like appearance [20]. From mutational mapping (Additional File 1 - Figure S1) it is notable that most mutations causing harmful or lethal phenotypes occur in close proximity to the active site, thereby affecting the enzyme functionality. Mutations distant from the active and binding sites cause minimal damage to the protein structure and activity, consequently resulting in mild phenotypes. However, a few mutations that are distant from the active site cause severe phenotypes due to their adverse effects on the enzyme structure and function (discussed later).

**Figure 3:**
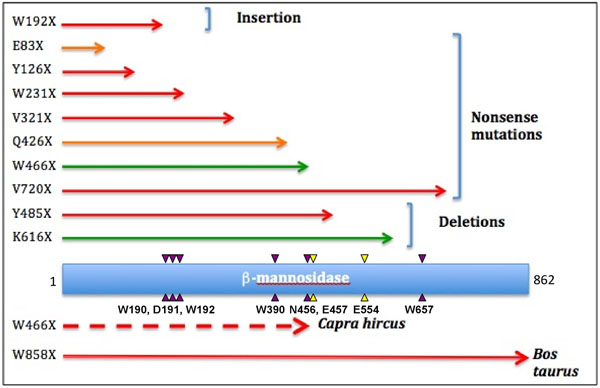
**Mapping truncations to the human β-mannosidase protein.** The extent of truncated mutants caused by nonsense, insertion and deletion mutations are shown as red, orange and green arrows, representing severe, moderate and mild phenotypes, respectively. Truncations in goat (broken arrow) and cow are shown below protein chain (blue rectangle). The purple and yellow arrow-heads represent the locations of the binding site residues and the catalytic nucleophiles, respectively. The corresponding single letter amino acid codes and ther residue numbers are given below the lower arrow-heads.

#### Most truncations cause severe phenotypes

Out of a total of 15 β-mannosidosis causing mutations identified up to date (Additional File 2 – Table S1) in three species: human, cow and goat, only two have been reported in ruminants. Both these mutations in ruminants are single base substitutions that introduced stop codons leading to truncations at the protein level (Figure 3). In Nubian goat, the active site of the enzyme was completely compromised by the W466X truncation (Figure 3), leading to lethal mannosidosis with neonatal onset [35,36]. In domestic cows, although the W858X truncation occurred on the N-terminal β-sheet resulting in almost the entire protein being intact (Figure 3; only 22 residues were missing), the animals still suffered deadly symptoms [8]. This highlights the significance of the role played by the last domain of β-mannosidase in enzyme stabilization, as suggested by Leipprandt et al. [8]. It is also notable that the enzyme chitobiase, the only exoglycosidase in the bi-directional catabolic pathway for *N-*acetyllactosaminic glycoproteins apart from β-mannosidase, is highly expressed and said to substitute for β-mannosidase activity in its absence or malfunctioning in human and rodents but not in ruminants [37]. This suggests that mutations that have an effect on the structure and function of β-mannosidase are likely to cause lethal phenotypes in those animals. However, human and mouse may be more tolerant for such mutations owing to the prominent presence of the enzyme chitobiase. 

**Figure 4:**
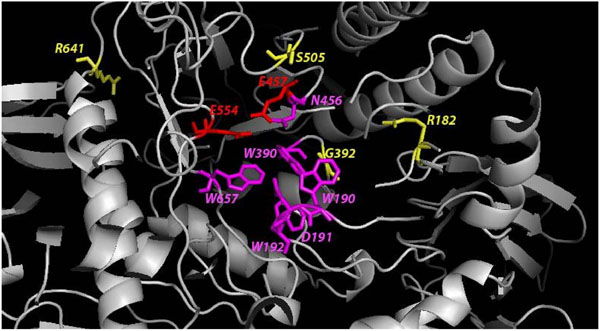
**Proximity of substitution mutations to β-mannosidase active site.** Amino acids are colored in red, magenta and yellow corresponding to the catalytic nucleophiles, ligand binding site residues and residues involved in substitutions, respectively. The active site residues and the residues involved in substutions are shown as sticks, while the rest of the protein is in ribbon representation. The model shown is that of WT human β-mannosidase.

In human, truncations make up approximately 70% of β-mannosidosis cases (9 out of 13 mutations). Most of these truncations caused the protein sequence to terminate well before the catalytic nucleophiles of the enzyme active site. As a result, they would completely demolish the enzyme activity. In fact, patients with Y126X [7], W192X [14], W231X [38], V321X [39] and Y485X [9] truncations showed typical severe phenotypes such as mental retardation, hearing loss and some degrees of angiokeratomas, with the age of onset ranging from infantile to 18 months [15,40]. Figure 3 provides an overview of the correlation between genotypic variations and phenotypic expressions amongst truncations obtained by mapping the extent of truncated protein in each case, caused by nonsense, insertion and deletion mutations within the *MANBA* gene sequence, to the human β-mannosidase protein chain. Generally, truncations before the β-mannosidase active site would result in improper functioning or non-functional protein, which subsequently leads to severe phenotypic expression. On the other hand truncations that lead to only a small segment of the protein chain being removed, are usually viable both in their genotypic and phenotypic expressions.

In some cases of β-mannosidosis however, the observed phenotypes were different from the theoretical consequences of the related mutations. One of the reasons for this conflict is the pattern of inheritance of the disease-associated mutations. Belidu *et al*. [9] reported a case, where the patient was heterozygous for both E83X and Q426X (Figure 3) mutations on different chromosomes. Yet, the patient only suffered moderate phenotypes, regardless of the total malfunctioning of the truncated proteins. Comparably, a Spanish woman was reported with mild mannosidosis, as she was a compound heterozygote for W466X truncation [10]. The K616X truncation results in a protein without W657, one of the active site residues, from the peptide sequence, but the individual only showed mild symptoms from the age of 12 as he was a heterozygote for the described mutation [10]. These variations in the genotypic and phenotypic expressions of the disease could also be attributed to the higher expression of the enzyme chitobiase in human as described earlier.

Similarly, V720X truncation resulted in almost an intact enzyme with full active site. Regardless, the mutation was reported to be responsible for severe clinical expression in the patient [41]. This is due to the fact that this truncation results in a protein without the entire last domain and half of the penultimate domain. Therefore, it causes improper domain organization leading to the destabilization of the enzyme fold and assembly. Hence, this truncation affects the enzyme functionality causing a severe phenotype. This observation sheds light on the potentially crucial role played by the last domain of β-mannosidase in enzyme stabilization in human as previously suggested for bovine β-mannosidase [8].

#### Substitutions affecting enzyme structure and function cause harmful phenotypes

**Figure 5:**
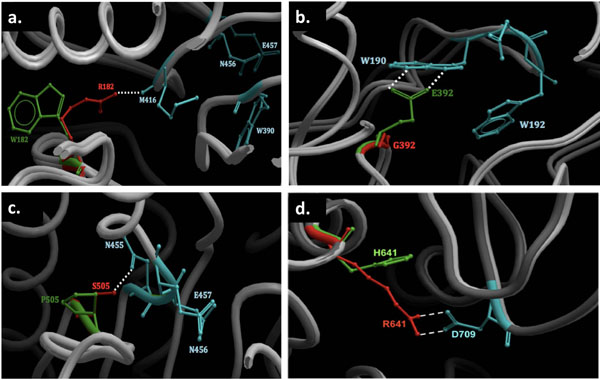
**The effect of substitutions on human β-mannosidase structure. a. R182W, b. G392E, c. S505P and d. R641H.** Sections where the substitutions occur on the WT and mutant human β-mannosidase structures are shown. The WT and mutated residues are shown in red and green, respectively. The residues affected by substitutions are coloured turquoise. All amino acids involved in the mutations and/or affected by the substitutions are in stick representation, while the segments of the protein backbone are shown in ribbon representations (light grey). The dotted lines signify the interactions between the amino acid residues.

Only 4 substitutions have been reported to date to be associated with β-mannosidosis. All the substitutions reported occur in human. The proximity of each mutation to the active site and properties of the amino acids involved, both contribute to the severity of the phenotype. The relative positions of these mutations with respect to the active site are visualized in Figure 4. In R182W substitution, replacement of the basic arginine by a neutral and aromatic tryptophan causes disruption of the dipole-dipole interaction between the side chain of R182 and the backbone of M416, as shown in Figure 5a. As a result, the TIM barrel structure that houses the enzyme active site is slightly destabilized and the enzyme activity is reduced [10]. However, since this mutation occurs away from the active site and the patient was a compound heterozygote for the same, the individual was reported with mild angiokeratoma corporis diffusum and no neurological involvement [10]. On the other hand, the G392E (Figure 5b) substitution changes the neutral glycine residue to an anionic glutamic acid [10] which forms an anion-quadrupole interaction with the electropositive ring edge of W190 (Figure 5). Consequently, the W190, D191 and W192 motif is dislocated from its optimal binding position. Hence, the enzyme activity is significantly reduced and the individual suffered from moderate symptoms [42]. In another case, replacement of a serine by a proline (S505P) causes disruption of the hydrogen bond between S505 and N455 (Figure 5c) which is located next to the catalytic nucleophile (E457) in the protein sequence (Figure 5c), thereby, disturbing the active site. In addition, introduction of a proline also causes distortion to the protein backbone. As a result, severe phenotypic expressions are observed in the affected patient [7].

**Figure 6:**
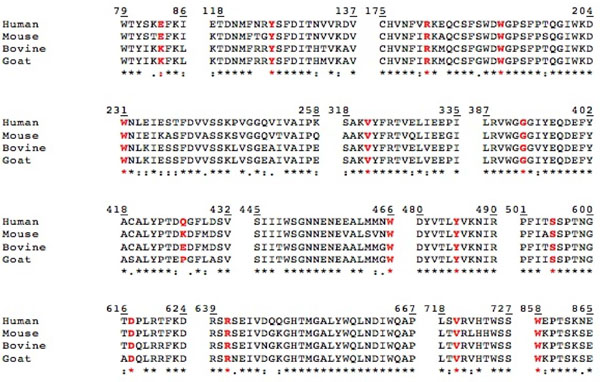
**Conservation of β-mannosidase mutational residues across the four WT sequences. **Multiple sequence alignment performed using ClustalX [26], shows a high level of sequence similarity, with conserved (*), conservatively substituted (:) and semi-conservatively substituted (.) residues. Sequence segments known to be mutational hot-spots are shown with the mutational residues highlighted in red.

In 2009, Sabourdy *et al.*[43] raised attention to another case, where the patient was diagnosed to be homozygous for an arginine to histidine substitution (R641H). The replacement of a positively charged arginine residue to that of a slightly positive histidine could rupture the potential salt bridge between R641 and D709 (Figure 5d) that exists in the human WT structure. This salt bridge could well be contributing towards the overall stability of the β-mannosidase protein fold. Hence, R641H substitution destabilizes the fold owing to the smaller and lesser charged histidine side-chain which cannot reach out to D709 to form a salt bridge (Figure 5d). Thus, this substitution affects the structure of the enzyme, thereby, significantly reducing enzyme activity resulting in a moderate phenotype. Also, R641 is conserved in most mammals (Figure 6), suggesting an important role of the residue in stabilizing the protein structure.

**Figure 7:**
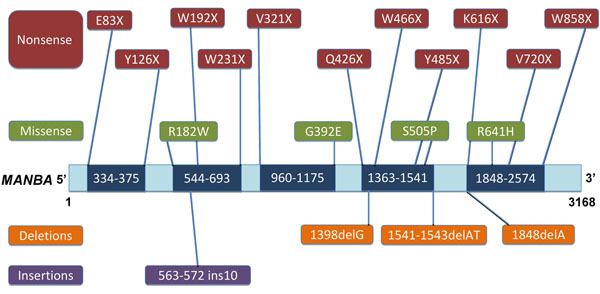
**Mapping inherited mutations in β-mannosidosis to the *MANBA* gene sequence.** Missense and nonsense mutations are shown above the gene sequence, with the specific amino acid change indicated, while insertions and deletions are shown below the gene sequence, numbered in terms of the bases affected. The dark blue areas on the *MANBA* gene sequence represent sequence-derived mutational hot-spots.

Analysis of our structural models laid the foundation for the correlation between the observed phenotype of each substitution mutation to their position and functional consequence. From Table 2 it is evident that mutations close to active site (severely disturbing the protein structure) and those that act to destabilize the fold directly affect the enzyme function leading to moderate or severe phenotypes. Where as, a mutation (R182W) away from the active site causes minimal damage to the enzyme structure and function leading to a mild or viable genotype and phenotype.

**Table 2:** Correlation of the effect of substitution mutations on protein folding and the observed phenotypic expression in β-mannosidosis. AS: Active site

Species	Mutated residue	Structural location	Effect on structure	Effect on phenotype
Human	R182W	Away from AS	Minor perturbation	Mild
Human	G392E	Close to AS	Disrupts the ligand binding site	Moderate
Human	S505P	Close to AS (direct interaction with AS)	Disturbs the catalytic nucleophile (E457)	Severe
Human	R641H	Away from AS	Destabilizes the fold	Moderate

### Prediction of potentially harmful mutations

Lysosomal β-D-mannosidase is an important component in the catabolic pathway for *N*-acetyllactosaminic glycoproteins [44]. Therefore, the protein structure is evolutionarily conserved across the species. Amino acid residues critical for enzyme stability and activity are well preserved, with only minor changes to suit different metabolic specificities in different species. Figure 2 shows the highly conserved residues or the components that form the enzyme active site. Therefore, any direct mutations or that associated with these residues could lead to serious consequences. Sedel *et al.*[14] have shown that insertion of 10 nucleotides at the 562-572 loci in the *MANBA* sequence, which encode the vital residue W192 (part of the W190, D191 and W192 motif), significantly demolishes the enzyme activity leading to a severe phenotype in the affected patient [14]. Moreover, mutations of the catalytic nucleophiles E457 and E554, located at the center of the active site, would completely demolish enzyme activity and hence could cause fatal phenotypes. Hence, we have extrapolated observed mutations from one species and mapped them to homologous positions in other organisms (Figure 6). Based on the proximity of these mutations to the enzyme active site and their co-location from different organisms, we have detected functional hot-spots for mutations likely to be involved in -mannosidosis across the four species (Figure 6).

Besides the functional hot-spots, mutational mapping also reveals high-risk segments on the β-mannosidase protein sequence. Figure 6 clearly shows that all the mutational residues, are highly conserved, with a small exception of E83 in human and mouse being replaced by K83 in ruminants and Q426 in human being replaced by K426 in mouse, E426 in domestic cow and P426 in goat. Our analysis reveals that Y126, W192, W231, V321, G392, Q426, Y485, S505, R641 and V720 are prime locations for β-mannosidosis mutations in human. Therefore, mutation of these residues could result in harmful disease phenotypes in mouse and ruminants. Truncation at W466 has reportedly resulted in a mild phenotype in human but causes a severe phenotypic effect in goat. Given the high level of conservation of this residue across the four species and keeping in mind the high and low levels of expression of the enzyme chitobiase amongst the species (as discussed earlier), if this mutation is identified in mouse and cow, it is likely to cause a mild and severe phenotype, respectively.

Similarly, truncation at W858 leads to a lethal phenotype in domestic cow. Hence, due to the crucial role played by the last domain of β-mannosidase in enzyme stabilization in cow [8] and the severe phenotype caused by the V720X truncation in human, W858X truncation could possibly cause severe β-mannosidosis in human, mouse and goat. Although R182 and K616 have only caused mild phenotypic expression in human and have not yet been detected in goat, cow and mouse, high level of sequential and positional similarity of these residues in all species suggests that they could be potential mutational hot-spots in goat, cow and mouse. Together with the active site amino acids, these disease-implicated residues form a set of hot-spots for β-mannosidosis prediction, which are useful for early detection of the disease.

### Mutational hot-spots in the *MANBA* gene

All inherited mutations known to cause β-mannosidosis were mapped onto the MANBA gene sequence based on their nucleotide position as listed in Additonal File 2 – Table S1. Although β-mannosidase mutations appear scattered when mapped onto the *MANBA* gene sequence, Figure 7 clearly shows mutational clusters in five distinct segments that vary in length, along the gene sequence. These clusters were identified by grouping mutations with relatively close nucleotide positions. The boundaries of these clusters were based on the nucleotide positions of the first and the last mutations in that particular group. These segments are hot-spots on the *MANBA* gene sequence that could most likely undergo harmful modifications due to high frequency of clinically observed mutations in these regions. These hot-spots range from nucleotide number 334-375, 544-693, 960-1175, 1363-1541 and 1848-2574, with nucleotide lengths varying from 42 to 727. The proximity of each hotspot segment to the active site is another factor that determines the probability of a harmful mutation occurring in that specific locus. 

Hot-spots 960-1175, 1363-1541 and 1848-2574 have the largest number of nucleotides (216, 179 and 727, respectively) amongst the five and are associated with the highest number of mutations causing β-mannosidosis. Therefore, it is inferred that the amino-acid residues encoded by the nucleotides within these hot-spots are prone to mutations. Hot-spots 544-693, 960-1175, 1363-1541 and 1848-2574, together code for all the active site residues. Hence, these segments could potentially harbour harmful mutations. Also, hot-spots 1363-1541 and 1848-2574 contain the genetic information that codes for the two catalytic nucleophiles (E457 and E554) of the enzyme. Hence, any inherited mutation identified within these two hot-spots could potentially cause more severe phenotypes than those identified elsewhere in the *MANBA* gene sequence.

## Conclusion

Although β-mannosidosis has been reported since 1980s, the underlying genotypic features have not been well studied. Clinically, typical symptoms considered associated with the disease are mental retardation, hearing loss, angiokeratomas, recurrent infection, skeletal and facial dysmorphism. However, the severity of phenotypic expression varies. While the disease is fatal in ruminants, it has varied phenotypes in human. This has been suggested to be a result of the high level of expression of another exoglycosidase enzyme called chitobiase in the human lysosomes [18,37]. Chitobiase acts on the reducing-end GlcNAc and removes it from the protein/carbohydrate component of N-linked glycoproteins [45]. In theory, chitobiase might be up-regulated in the absence of β-mannosidase to act as a replacement enzyme. However, a combined study of β-mannosidase and chitobiase would be interesting to help better understand β-mannosidosis and its degree of phenotypic expression.

Our comprehensive study has revealed a genotype–phenotype correlation in β-mannosidosis. Since the components of β-mannosidase active site span across the length of the protein chain, homozygous truncations are very likely to result in a dysfunctional enzyme leading to severe phenotypes. The effects of substitutions are also related to their proximity to the enzyme active site. In particular, the closer the mutations are to the active site, the more severe the phenotypes would be. The pattern of inheritance for the mutations is also an importance factor that affects the clinical expression of the disease. Unlike β-mannosidosis, where mutations are homozygous with distinct expression, β-mannosidosis can be either heterozygous or homozygous, which significantly reduces the chances of identification in patients with mild mutations. This poses a major restriction in studying β-mannosidosis. Therefore, a total of 15 mutations reported in three decades (Additional File 2 – Table S1) provide a reasonable dataset for analysis.

Our analysis reveals that the mutations are scattered over the entire length of the MANBA gene, presenting us with five distinct mutational hot-spots. The identification of these regions has not only provided us the basis for predicting the severity of a particular mutation, it has also helped us in forecasting the residues that could most likely undergo mutations based on their location on the gene. Moreover, much like many other lysosomal disorders, a high amount of mutational heterogeneity is observed in β-mannosidosis. This property of β-mannosidosis makes it comparable to other lysosomal disorders. We have also demonstrated a predictive approach for detecting likely β-mannosidosis by extrapolating observed mutations from one species to homologous positions in other organisms based on the proximity of the mutations to the enzyme active site, mutational co-location from different organisms (human, cow and goat) and orthologous positions in the profile alignment (Additional File 1 – Figure S1) of the WT β-mannosidase sequences from four species (human, cow, goat and mouse).

From our investigation, we infer that β-mannosidosis could be dealt through gene therapy rather than drug/inhibitor design since lysosomal β-mannosidase is a cardinal enzyme and all mutations observed affect it’s functionality. By highlighting the effect of disease-implicated mutations on enzyme/protein structure, this study provides a basis for understanding the molecular determinants responsible for phenotypic variations. This study could be extended to other inherited diseases with potential applications in developing therapies to combat them. Should a larger set of β-mannosidosis associated mutations become available in the future, it would be very interesting to apply this structural analysis procedure to authenticate our genotype-phenotype correlation. While this study provides essential knowledge of the effect of mutations on β-mannosidase structure and function by analyzing its static structures, such studies will no doubt benefit from either experimental or computational analysis of the enzyme structural dynamics in order to gain an in-depth understanding of the underlying causes of β-mannosidosis. Furthermore, there will also be the possibility that while mutations may be detected, they may not result in any diseased phenotype because upon combinations, mutations may compensate for the deleterious effects of each other.

## Competing interests

The authors declare that they have no competing interests.

## Authors’ contributions

TH carried out the computational simulation studies. TH and JMK drafted the manuscript. TH, JMK and SR participated in the design of the study and interpretation of data. SR developed the project and finalized the manuscript. All authors have read and approved the final manuscript.

## Supplementary Material

Additional File 1 **Figure S1. Mapping inherited mutations in β-mannosidase onto its secondary structure.** Profile alignment of the four WT sequences to the template (PDB ID: 2JE8) sequence is shown. All the sequences are numbered accordingly. The secondary structural elements of the enzyme were mapped onto the profile alignment prior to mutational mapping. The catalytic nucleophiles of the enzyme are highlighted in dark green and the binding site residues are in light green. Residues in blue belong to the TIM barrel. Mutations are positioned on the secondary structure above the mutational residue from the alignment. Truncations are shown in red and substitutions are in pink. Click here for file

Additional File 2 **Table S1. List of inherited mutations in β-mannosidase from human (H), goat (G) and cow (C).** The positional changes in the *MANBA* gene sequence and their consequences on the protein structure are listed. Also listed are the typical phenotypic effects (disease symptoms) of the mutation and the age of onset amongst the individuals. Click here for file
