# Supplementary material for: A comparative structural bioinformatics analysis of inherited mutations in β-D-Mannosidase across multiple species reveals a genotype-phenotype correlation
Source: BMC Genomics. 2011 Nov 30;12(Suppl 3):S22. doi: 10.1186/1471-2164-12-S3-S22 (PMC3333182; doi:10.1186/1471-2164-12-S3-S22)
Supplement: Additional File 1 — Figure S1. Mapping inherited mutations in β-mannosidase onto its secondary structure. Profile alignment of the four WT sequences to the template (PDB ID: 2JE8) sequence is shown. All the sequences are numbered accordingly. The secondary structural elements of the enzyme were mapped onto the profile alignment prior to mutational mapping. The catalytic nucleophiles of the enzyme are highlighted in dark green and the binding site residues are in light green. Residues in blue belong to the TIM barrel. Mutations are positioned on the secondary structure above the mutational residue from the alignment. Truncations are shown in red and substitutions are in pink. [file 1471-2164-12-S3-S22-S1.pdf]

Additional File 1

A comparative structural bioinformatics analysis of inherited mutations in β-D-Mannosidase across multiple species reveals a genotype-phenotype correlation

Thi Huynh, Javed Mohammed Khan and Shoba Ranganathan

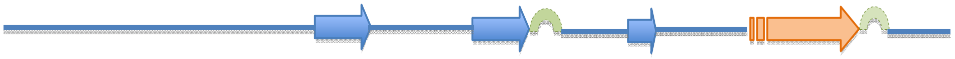

```
Human       1 MRLHLLLLLALCGAGTTAAELSYSLRGNWSICNGNGSLELPGAVPGCVHSALFQQGLIQD
Bovine      1 MLLRLLLLLAPCGAGFATKVVSISLRGNWKIHSGNGSLQLPATVPGCVHSALFNKRIIKD
Goat        1 MLLRLLLLLAPCGAGFATEVVSISLRGNWKIHNGNGSLQLPAAVPGCVHSALFNKRIIKD
Mouse       1 MHLHLLLILALFRAGCVVAGPSYSLSGSWRVSNGNGSLELPATVPGYVHSALHQHGLIQD
2JE8          --------------GNDTSEVMLLDTG-WEFSQSGTEKWMPATVPGTVHQDLISHELLPN
```

E83X

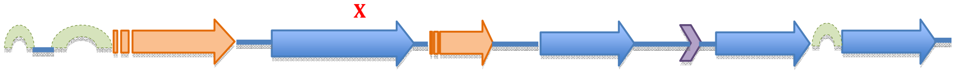

```
Human      61 SYYRFNDLNYRWVSLDNWTYSKEFKIPFEISKWQKVNLILEGVDTVSKILFNEVTIGETD
Bovine     61 PYYRFNNLDYRWIALDNWTYIKKFKLHSDMSTWSKVNLVFEGIDTVAVVLLNSVPIGKTD
Goat       61 PYYRFNNLDYRWIALDNWTYIKKFKLHSDMSEWNKVNLVFEGIDTVAVVLLNSVPIGKTD
Mouse      61 PYYRFNDLNYRWISLDNWTYSTEFKIPFNLSEWQKVKLIFDGVDTVAEILFNNVTIGKTD
2JE8       46 PFYGMNEKKIQWVENEDWEYRTSFIVSEEQLNRDGIQLIFEGLDTYADVYLNGSLLLKAD
```

Y126X

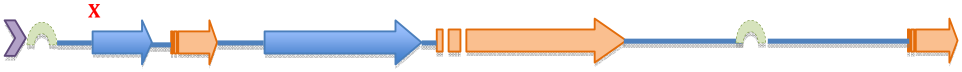

```
Human     121 NMFNRYSFDITNVVR-DVNSIELRFQSAVLYAAQQSKAHTRYQVPPDCPPLVQKGECHVN
Bovine    121 NMFRRYSFDITHTVK-AVNIIEVRFQSPVVYANQRSERHTAYWVPPNCPPPVQDGECHVN
Goat      121 NMFRRYSFDITHMVK-AVNIIEVRFQSPVIYANQRSERHTAYWVPPNCPPPVQDGECHVN
Mouse     121 NMFTGYSFDITNVVK-DVNSLKLQFRSAVQYAECQSKAHTSYRVPPECPPVEQKGECHVN
2JE8      106 NMFVGYTLPVKSVLRKGENHLYIYFHSPIRQTLPQYASN-GFNYPADN---DHHEKHLSV
```

R182W W192X W231X

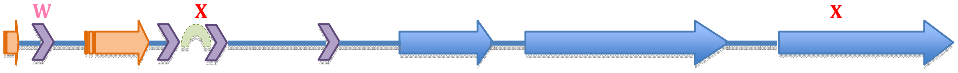

```
Human     180 FVRKEQCSFSWDWGPSFPTQGIWKDVRIEAYNICHLN-YFTFSPIYDKSAQEWNLEIEST
Bovine    180 FIRKMQCSFGWDWGPSFPTQGIWKDVRIEAYNVCHLN-YFMFTPIYDNYMKTWNLKIESS
Goat      180 FIRKMQCSFGWDWGPSFPTQGIWKDVRIEAYNICHLN-YFMFTPIYDNYMETWNLKIESS
Mouse     180 FIRKAQCSFSWDWGPSFPSQGIWKDVRIEAYNIAHLD-YLTFLPVYDNASQAWNIEIKAS
2JE8      162 FSRKAPYSYGWDWGIRMVTSGVWRPVTLRFYDIATISDYYVRQLSLTDENARLSNELIVN
```

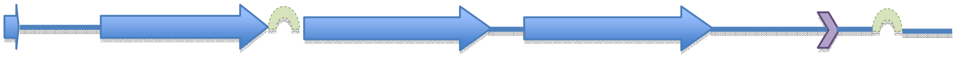

```
Human     239 FDVVSSKPVGGQVIVAIP-KLQTQQTYSIELQPGKRIVELFVNISKNITVETWWPHGHGN
Bovine    239 FDVVSSKLVSGEAIVAIP-ELNIQQTNNIELQHGERTVELFVKIDKAIIVETWWPHGHGN
Goat      239 FDVVSSKLVSGEAIVAIP-ELNIQQRNNIELRHGERTVKLFVKIDKAVIVETWWPHGHGN
Mouse     239 FDVASSKSVGGQVTVAIP-QLKTQQTNDIELQQEQRIVKLLVKIRKDVAVETWWPRGHGN
2JE8      222 QIVPQKIPAEVRVNVSLNGTTVTEVKQQVTLQPGINHITLPAEVTNPVR---WMPNGWGT
```

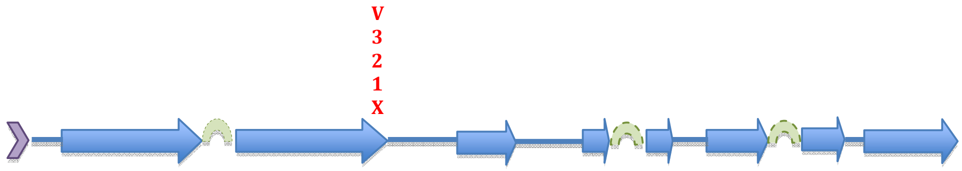


```
Human    298 QTGYNMTVLFELDGGLNIEKSAKVYFRTVELIEEPIKGSPGLSFYFKINGFPIFLKGSNW
Bovine   298 QTGYNMSVIFELDGGLRFEKSAKVYFRTVELVEEPIQNSPGLSFYFKINGLPIFLKGSNW
Goat     298 QTGYDMTVTFELDGGLRFEKSAKVYFRTVELVEEPIQNSPGLTFYFKINGLPIFLKGSNW
Mouse    298 QTGYNMTILFALDGGLKIEKAAKVYFRTVQLIEEGIKGSPGLSFYFKINGLPIFLKGSNW
2JE8     279 PTLYDFSAQIACGDRIVAEQSHRIGLRTIRVVNE--KDKDGESFYFEVNGIPMFAKGANY
```

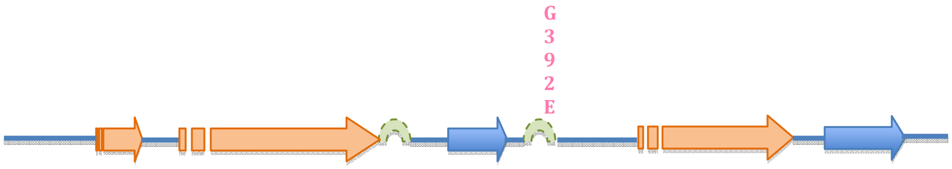


```
Human    358 IPADSFQDRVTSELLRLLLQSVVDANMNTLRVWGGGIYEQDEFYELCDELGIMVWQDFMF
Bovine   358 IPADSFQDRVTSAMLRLLLQSVVDANMNALRVWGGGVYEQDEFYELCDELGIMIWQDFMF
Goat     358 IPADSFQDRVTSDMLRLLLQSVVDANMNALRVWGGGIYEQDEFYELCDELGIMIWQDFMF
Mouse    358 IPADSFQDKVTSDRLQLLFQSVVDANMNTLRVWGGGIYEQDEFYALCDELGIMVWQDFMF
2JE8     337 IPQDALLPNVTTERYQTLFRDMKEANMNMVRIWGGGTYENNLFYDLADENGILVWQDFMF
```

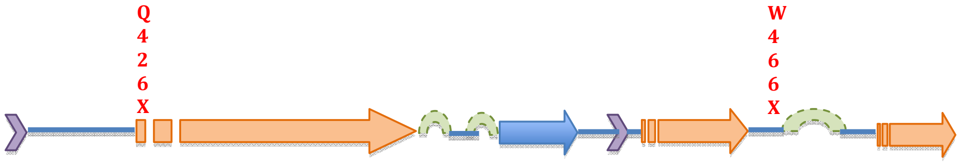


```
Human    418 ACALYPTDQGFLDSVTAEVAYQIKRLKSHPSIIIWSGNNENEEALMMNWYHIS--FTDRP
Bovine   418 ACALYPTDKDFMDSVREEVTHQVRRLKSHPSIITWSGNNENEAALMMGWYDTK--PGYLQ
Goat     418 ACALYPTDEDFMDSVREEVTHQVRRLKSHPSIITWSGNNENEAALMMGWYDTK--PGYLH
Mouse    418 ASALYPTEPGFLASVRKEVTYQVRRLKSHPSIIIWSGNNENEVALSVNWFHVN--PRDMK
2JE8     397 ACTPYPSDPTFLKRVEAEAVYNIRRLRNHASLAMWCGNNEILEALKYWGFEKKFTPEVYQ
```

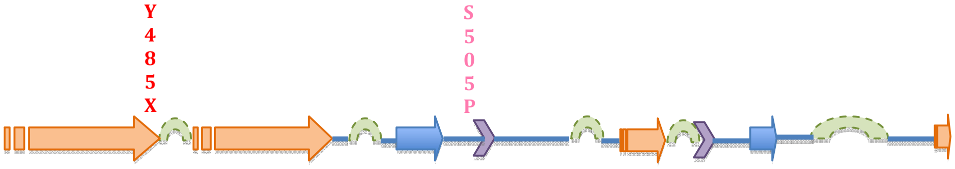


```
Human    476 IYIKDYVTLYVKNIRELVLAGDKSRPFITSSPTNGAETVAEAWVSQNPN--SNYFGDVHF
Bovine   476 TYIKDYVTLYVKNIRTIVLEGDQTRPFITSSPTNGAKTIAEGWLSPNPY--DLNYGDVHF
Goat     476 TYIKDYVTLYVKNIRTIVLEGDQTRPFIISSPTNGAKTTAEGWLSPNPY--DLNYGDVHF
Mouse    476 TYIDDYVTLYVKNIRKIVLSEDKSRPFIASSPTNGMKTMEEGWISYDPY--SIQYGDIHF
2JE8     457 GLMHGYDKLFRELLPSTVKEFDSDRFYVHSSPYLANWGRPESWGTGDSHNWGVWYGKKPF
```

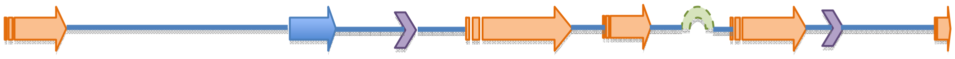

```
Human    534 YDYISDCWNWKVFPKARFASEYGYQSWPSFSTLEKVSSTEDWSFNSKFSLHRQHHEGGNK
Bovine   534 YDYVSDCWNWRTFPKARFVSEYGYQSWPSFSTLEKVSSEEDWSYRSSFALHRQHLINGNN
Goat     534 YDYMSDCWNWRTFPKARFVSEYGYQSWPSFSTLEKVSSEEDWSYESSFALHRQHLINGNS
Mouse    534 YNYADDCWNWKIFPKARLVSEYGYQSWPSFSTLEKVSSQEDWAYNSRFSLHRQHHEDGNH
2JE8     517 ESLDTDLP--------RFMSEFGFQSFPEMKTIAAFAAPEDYQIESEVMNAHQKSSIGNS
```

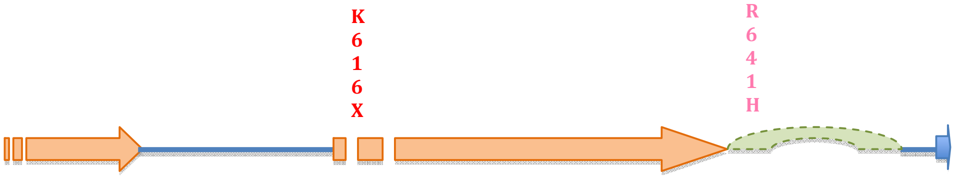


```
Human     594  QMLYQAGLHFKLPQSTDPLRTFKDTIYLTQVMQAQCVKTETEFYRRSRSEIVDQQGHTMG
Bovine    594  EMLHQIELHFKLPNSTDQLRRFKDTLYLTQVMQAQCVKTETEFYRRSRSEIVNGKGHTMG
Goat      594  EMLQQIELHFKLPNSADQLRRFKDTLYLTQVMQAQCVKTETEFYRRSRNEIVDGKGHTMG
Mouse     594  QMLHQVKMHFKLPQGTDPLRTFKDTIYLTQVMQAQCIKTETEFYLRSRSEIVDGKGHTMG
2JE8      569  LIRTYMERDYIIPES------FEDFVYVGLVLQGQGMRHGLEAHRRNRP-------YCMG
```

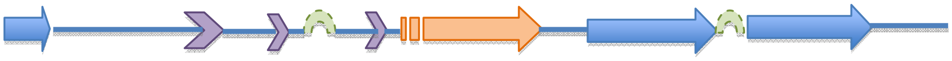

```
Human     654  ALYWQLNDIWQAPSWASLEYGGKWKMLHYFAQNFFAPLLPVGFENENTFYIYGVSDL-HS
Bovine    654  ALYWQLNDIWQAPSWSSLEYGGKWKMLHYFARHFFAPLLPVGFEDKDMLFIYGASHL-HS
Goat      654  ALYWQLNDIWQAPSWSSLEYGGKWKMLHYFARRFFAPLLPVGFEDKDVLFIYGVSDL-PS
Mouse     654  ALYWQLNDIWQAPSWASLEYGGKWKMLHYFARRFFAPLLPVGFEDEGVFYVYGVSDL-HK
2JE8      616  TLYWQLNDSWPVVSWSSIDYYGNWKALHYQAKRAFAPVLINPIQQNDSLSVYLISDRLDT
```

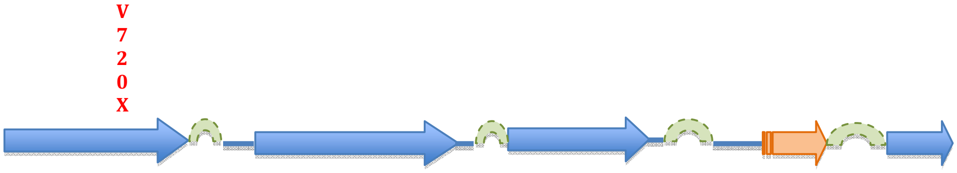


```
Human     713  DYSMTLSVRVHTWSSLEPVCSRVTERFVMKGGEAVCLYEEPVSELLRRCGNCTRESCVVS
Bovine    713  DQQMMLTVRVHTWSSLELVCSESTNPFVIKAGESVLLYTKPVPELLKGCPGCTRQSCVVS
Goat      713  DHQMMLTVRVHTWSSLELVCSELTNPFVMKAGESVVLYSKPVPELLKGCPGCTRQSCVVS
Mouse     713  DHHTQLTVRLHHWSSPKPLCSLVNSSIVVKAGEAVVLFQMPVSELLKRCRGCTRETCVVS
2JE8      676  MEQMTLEMKVVDFDGKTLGKKIQVHSLEVPANTSKCVYRAKLDGWLT-PEDCRRS--FLK
```

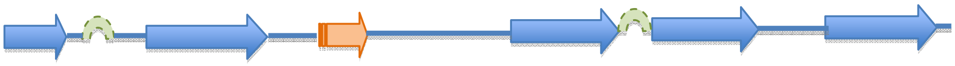

```
Human     773  FYLSADHELLSPTNYHFLSSPKEAVGLCKAQITAIISQQGDIFVFDLETSAVAPFVWLDV
Bovine    773  FYLSTDGELLSPINYHFLSSLKNAKGLHKANITATISQQGDTFVFDLKTSAVAPFVWLDV
Goat      773  FYLSTDGELLSPINYHFLSSLKNAKGLHKANITATISQQGNTFVFDLKTSAVAPFVWLDV
Mouse     773  FYFSTDKELFSPTNYHFLSSLKDAKGLLEANITVNISQKGNVFVFDLETSAVAPFVWLDV
2JE8      733  LILKDKSGHQVAESVHFFRKTKDLQ-LPPTSVSYQMKQTDGKCELTLFSSMLAKDIFIET
```

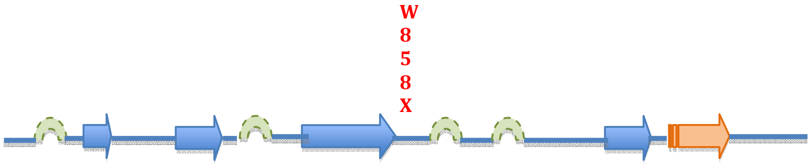


```
Human     833  GSIPGRFSDNGFLMTEKTRTILFYPWEPTSKNELEQSFHVTSLTDIY----
Bovine    833  GSIPGRFSDNGFLMTEKTRTVFFYPWKPTSKSELEQSFHVTSLADTY----
Goat      833  GSIPGRFSDNGFLMTEKTRTVFFYPWKPTSKSELEQSFHVTSLADTY----
Mouse     833  GSIPGRFSDNGFLMIRKKLSVLFYPWKPTSKSELQQAFSVTSLTDTY----
2JE8      792  PLQGARYSDNFFDLLPGERKKVIIT-SPRIKKGEELPVNIKHIRETYKEHH
```

Figure S1. Mapping inherited mutations in β-mannosidase onto its secondary structure. Profile alignment of the four WT sequences to the template (PDB ID: 2JE8) sequence is shown. All the sequences are numbered accordingly. The secondary structural elements of the enzyme were mapped onto the profile alignment prior to mutational mapping. The catalytic nucleophiles of the enzyme are highlighted in dark green and the binding site residues are in light green. Residues in blue belong to the TIM barrel. Mutations are positioned on the secondary structure above the mutational residue from the alignment. Truncations are shown in red and substitutions are in pink.
