# Supplementary material for: A comparative structural bioinformatics analysis of inherited mutations in β-D-Mannosidase across multiple species reveals a genotype-phenotype correlation
Source: BMC Genomics. 2011 Nov 30;12(Suppl 3):S22. doi: 10.1186/1471-2164-12-S3-S22 (PMC3333182; doi:10.1186/1471-2164-12-S3-S22)
Supplement: Additional File 2 — Table S1. List of inherited mutations in β-mannosidase from human (H), goat (G) and cow (C). The positional changes in the MANBA gene sequence and their consequences on the protein structure are listed. Also listed are the typical phenotypic effects (disease symptoms) of the mutation and the age of onset amongst the individuals. [file 1471-2164-12-S3-S22-S2.pdf]

Additional File 2

A comparative structural bioinformatics analysis of inherited mutations in β-D-Mannosidase across multiple species reveals a genotype-phenotype correlation

Thi Huynh, Javed Mohammed Khan and Shoba Ranganathan

Table S1. List of inherited mutations in β-mannosidase from human (H), goat (G) and cow (C). The positional changes in the *MANBA* gene sequence and their consequences on the protein structure are listed. Also listed are the typical phenotypic effects (disease symptoms) of the mutation and the age of onset amongst the individuals.

S. No.	Species	Sequence change		Age of onset	Disease symptoms	Reference
		DNA	Protein			
1	H	334G>T	E83X	5 years	Hearing loss, mental retardation and Angiokeratomas	[3, 9]
2	H	1363C>T	Q426X			
3	H	375A>G	Y126X	Infantile onset	Speech impairment, mental retardation, hyperactive behavior, facial dysmorphoism and respiratory infections	[7, 40]
4	H	1513T>C	S505P			
5	H	544C>T	R182W	24 years	Angiokeratoma, slight deafness and abdominal pain	[10]
6	H/G	1398G>A/1398delG	W466X	24 years/ Neonatal onset	Angiokeratoma, slight deafness and abdominal pain/Inability to rise, intention tremors, facial dysmorphism and carpal contractures	[10]/[16, 35]
7	H	563-572dup10	W192X	18 months	Delay in speech acquisition, attention-deficit/hyperactivity disorder, aggressiveness compatible with Gilles de la Tourette syndrome, recurrent upper airway infections and hearing loss	[14]
8	H	693G>A	W231X	Juvinile onset	Mental retardation since childhood, aggressive behavior, deafness, recurrent erysipelas, mild renal failure and multiple angiokeratomas	[38]
9	H	1175G>A	G392E	12 years	Angiokeratoma and scantly communicative	[10, 15, 41]
10	H	1848delA	K616X			
11	H	1541-1543delAT	Y485X	15 months	Speech development problems, recurrent respiratory tract infections, hearing loss, motor and mental retardation (age 5), hyperactivity and aggressive behavior (age 18), facial dysmorphism and skeletal deformation	[9]

S. No.	Species	Sequence change		Age of onset	Disease symptoms	Reference
		DNA	Protein			
12	H	960+1A>G	V321X	2 years	Hearing loss, mental retardation, developmental disorders, hypertension, chronic renal failure and numerous angiokeratomas	[38, 39]
13	H	2015-2A>G	V720X	14 months	Severe psychomotor retardation, bone deformities, facial dysmorphism, macroglossia, gingivial hyperplasia, umbilical hernia, recurrent skin and respiratory infections, hypotonia and aggressive behavior	[3, 43]
14	H	1922G>A	R641H	4 years	Mental retardation, loss of cognitive function, spastic tetraparesis, cerebellar ataxia, visual and hearing deficits at age 12, tetraplegia, dysphagia and dysarthria at age 26	[42]
15	C	2574G>A	W858X	Neonatal onset	Inability to rise with intention tremors, hidebound skin, slightly domed calvaria, slight prognathism and narrow palpebral fissures	[8, 36]
